# A Novel Quantitative Method for Tooth Grinding Surface Assessment Using 3D Scanning

**DOI:** 10.3390/diagnostics11081483

**Published:** 2021-08-16

**Authors:** Benedikt Sagl, Ferida Besirevic-Bulic, Martina Schmid-Schwap, Brenda Laky, Klara Janjić, Eva Piehslinger, Xiaohui Rausch-Fan

**Affiliations:** 1 Center of Clinical Research, University Clinic of Dentistry, Medical University of Vienna, 1090 Vienna, Austria; brenda.laky@meduniwien.ac.at (B.L.); klara.janjic@meduniwien.ac.at (K.J.); xiaohui.rausch-fan@meduniwien.ac.at (X.R.-F.); 2 Division of Prosthodontics, University Clinic of Dentistry, Medical University of Vienna, 1090 Vienna, Austria; ferida.besirevic-bulic@meduniwien.ac.at (F.B.-B.); martina.schmid-schwap@meduniwien.ac.at (M.S.-S.); eva.piehslinger@meduniwien.ac.at (E.P.)

**Keywords:** sleep bruxism, digital dentistry, diagnostic bruxism splint

## Abstract

Sleep bruxism is an oral parafunction that involves involuntary tooth grinding and clenching. Splints with a colored layer that gets removed during tooth grinding are a common tool for the initial diagnosis of sleep bruxism. Currently, such splints are either assessed qualitatively or using 2D photographs, leading to a non-neglectable error due to the 3D nature of the dentition. In this study we propose a new and fast method for the quantitative assessment of tooth grinding surfaces using 3D scanning and mesh processing. We assessed our diagnostic method by producing 18 standardized splints with 8 grinding surfaces each, giving us a total of 144 surfaces. Moreover, each splint was scanned and analyzed five times. The accuracy and repeatability of our method was assessed by computing the intraclass correlation coefficient (ICC) as well reporting means and standard deviations of surface measurements for intra- and intersplint measurements. An ICC of 0.998 was computed as well as a maximum standard deviation of 0.63 mm^2^ for repeated measures, suggesting an appropriate accuracy of our proposed method. Overall, this study proposes an innovative, fast and cost effective method to support the initial diagnosis of sleep bruxism.

## 1. Introduction

Traditionally, bruxism is defined as an oral parafunction involving involuntary tooth grinding and clenching [1]. Moreover, a distinction is made between awake and sleep bruxism, which potentially have different origins and pathophysiology [2]. Bruxism is a possible risk factor for different pathologies and can lead to severe abrasion of teeth, tooth hypermobility, masticatory muscle pain, headache, periodontal tissue damage as well as temporomandibular joint (TMJ) pain. Most people will go through phases of tooth grinding or clenching during the course of their lifetime [3] with studies reporting approximately 5–13% of adults as frequent tooth grinders [4,5,6].

Diagnosis of bruxism is a challenging task due to its involuntary nature. Initial assessment often relies on reports of tooth grinding sounds and symptoms such as flattened teeth, which already imply a rather late time of diagnosis. The American Academy of Sleep Medicine defined diagnostic criteria for sleep bruxism, which involve the occurrence of abnormal tooth wear, associated sounds and jaw discomfort [5]. A polysomnographic (PSG) investigation, including video, audio as well as a multitude of different respiratory, muscular and other parameters, is generally seen as the gold standard for a definitive diagnosis of sleep bruxism [7]. Since PSG is very expensive and time consuming for the patient, many studies have used electromyography (EMG) [8] devices to measure masticatory muscle activity during sleep, investigating rhythmic masticatory muscle activity (RMMA), which is a diagnostic sign of sleep bruxism [9,10]. Another approach using an instrumented splint to measure peaks in bite force has been proposed previously [11].

While EMG gives reliable information on RMMA occurrence and, as a consequence, helps with detecting bruxism [10], portable EMG devices are still rather expensive and most clinics do not own enough devices to easily use them for every potential patient. A previously proposed simple and cost-effective tool for bruxism diagnosis is a colored splint to monitor tooth contact during sleep. The first reports of this method go back to the 1970s [12,13]. The proposed splint consists of four colored layers comprising an overall thickness of 0.51 mm. During grinding of the teeth, one or multiple, depending on the amount of grinding force, colored layers are ground off, revealing information on occlusal contact areas. More recently a semi-automatic method to analyze such splints has been published [14,15]. The method uses standardized pictures to measure the abraded area in a 2D projection but neglects the 3D nature of the tooth shape. Another comparable product was developed by a group at the Kanagawa Dental College [16]. While their splint only has a single colored layer, reducing the diagnostic information on bruxing force, it is very thin (0.1 mm thickness), which potentially limits the alteration of muscular activity during sleep caused by the splint [17]. To the best of our knowledge, analysis of this tool has also solely focused on quantitative assessments of occlusal grinding patterns in 2D projections (photographs) [18,19,20,21]. All analysis methods that rely on 2D projections infer an error, which increases with the angle between the projection plane and the tooth facet. With the advance of digital dentistry and improvements in the quality as well as the accessibility of 3D scanning devices, a logical next step would be the digitalization of occlusal splints and the detailed diagnostic analysis of the occlusal contacts using 3D mesh analysis approaches.

Consequently, the presented study proposes a novel method for the semi-automated 3D analysis of colored occlusal splints for the diagnostic investigation of tooth contacts in the context of bruxism. This method has the potential to gather more accurate information on nocturnal occlusal contacts in an easy and reliable fashion, helping clinicians to collect the information necessary for bruxism diagnosis, while only using equipment accessible in a dental practice.

## 2. Materials and Methods

**Figure 1:**
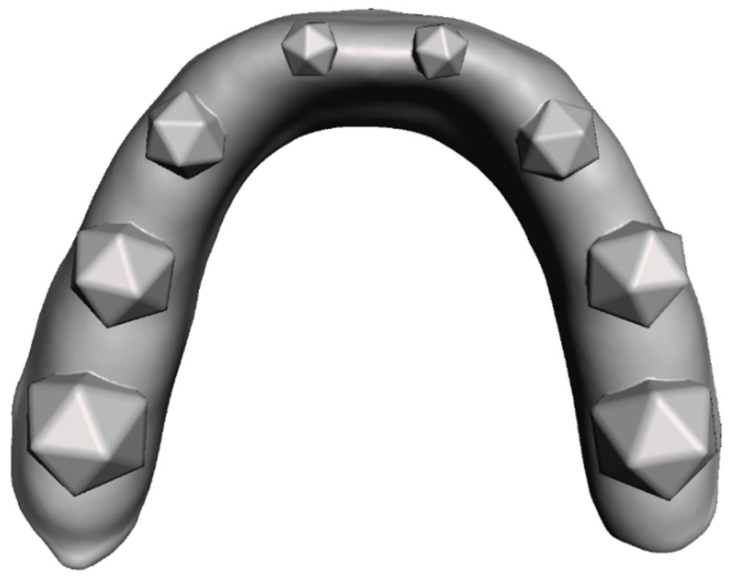
Top view of the 3D model created in the Meshmixer toolkit.

To test and validate our diagnostic method a model consisting of an idealized gingiva arch with a total of 8 embedded icosahedrons was designed using the Autodesk^®^ Meshmixer toolkit (Autodesk, San Rafael, CA, USA) (Figure 1).

To later test the performance of the presented method for different sizes of grinding surfaces, the geometrical bodies varied in size. The triangular surfaces of the icosahedrons’ faces decreased from posterior to anterior, with respective triangle heights of 5 mm, 4 mm, 3 mm and 2 mm. The base model was produced with an additive manufacturing approach using a Formlabs^®^ Form 2 printer (Formlabs, Somerville, MA, USA) and the Formlabs^®^ Dental LT Clear V1 resin (Formlabs, Somerville, MA, USA). The model was used in combination with a pressure molding device (Biostar^®^, Scheu Dental, Iserlohn, Germany) to produce the splints themselves from a dedicated pressure molding foil with one red-colored side and a thickness of 0.1 mm (Bruxchecker^®^, Scheu Dental, Iserlohn, Germany). After production the splints are relatively translucent and normally turn opaque in the patient’s mouth. To get the same effect in vitro, we submerged the finished splints in water with some added toothpaste for 6 h. After this step the splints showed surface opaqueness comparable to clinical splints.

**Figure 2:**
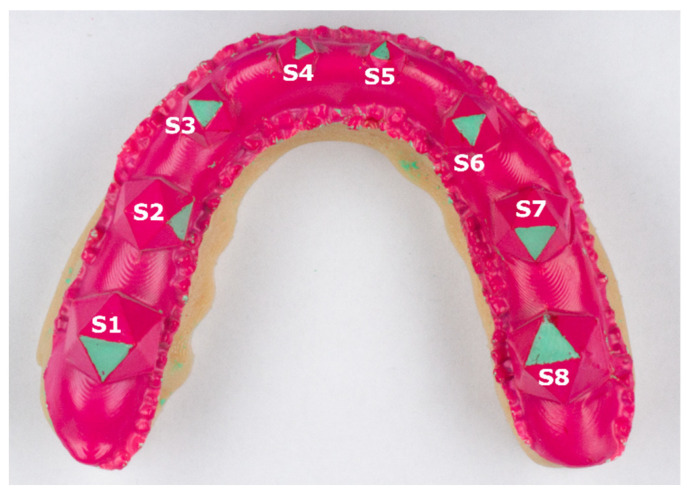
Example splint after “grinding surface” preparation and powder spraying. S1 to S8 depict the respective grinding surfaces.

To simulate tooth grinding, one triangle per icosahedron was prepared using a KaVo K4 handpiece (KaVo Dental, Biberach an der Riß, Germany) and the red layer was ground off to leave the respective surface transparent. Processed triangles varied between splints and were used to test the performance of our method for different surface angles. Scanning of the transparent surfaces lead to rather severe 3D reconstruction artifacts—consequently, we spray-painted the inside of the splint using a colored (green) powder spray (Occlu^®^Spray Plus, Hager & Werken, Duisburg, Germany) (Figure 2).

After preparation, splints were scanned using an optical 3D scanner (Primescan^™^ AC, Dentsply Sirona, Bensheim, Germany) and mesh files were exported as .ply files including vertex position as well as vertex color information. To check for intrascan accuracy of the method, each splint was scanned 5 times. Meshes were imported into the Meshmixer software toolkit (version 3.4) and the “grinding surfaces” were segmented using a semi-automatic method. For this purpose, an initial vertex inside the grinding surface was selected and the selection was expanded using a similarity measure of vertex color for the abraded areas. The abraded areas were green and the rest of the splint remained red. The surface area of each grinding facet was recorded for 18 splints for 5 repeated measurements, giving 90 scans and 720 grinding surfaces. A detailed description of our software workflow can be found in Appendix A.

Intraclass correlation coefficient (ICC) over the 5 repeated measures was evaluated and an analysis of variance (ANOVA) of repeated scans of the same physical splint was performed. To better describe the grinding surfaces we moreover reported the maximum, minimum, mean and relative standard deviations over the repeated measures for each grinding surface. Additionally, to showcase the differences in results computed using a 2D projection approach with respect to the proposed 3D method, all 18 splints were photographed using a standardized set-up and grinding areas were segmented in 2D using ImageJ (https://imagej.nih.gov/ij, [22]). We report mean surface areas and standard deviations for each grinding surface for both measurement methods and compared 2D photographs to 3D scans using an independent-samples *t*-test. Statistical assessment was performed using IBM SPSS Statistics 26^®^ (IBM, Armonk, NY, USA).

## 3. Results

**Figure 3:**
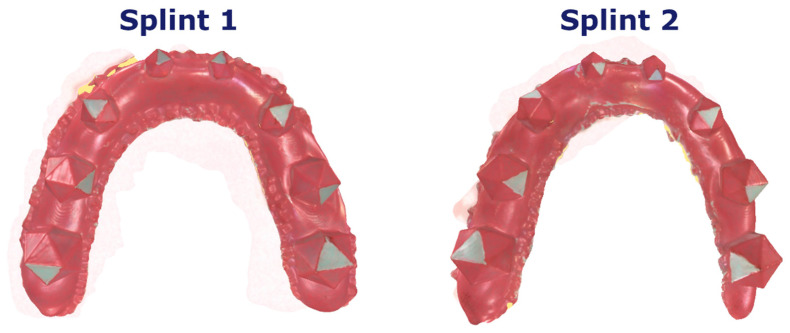
Example scans of two out of the 18 different splints; different combinations of triangles were prepared for each splint to investigate different surface angles.

**Figure 4:**
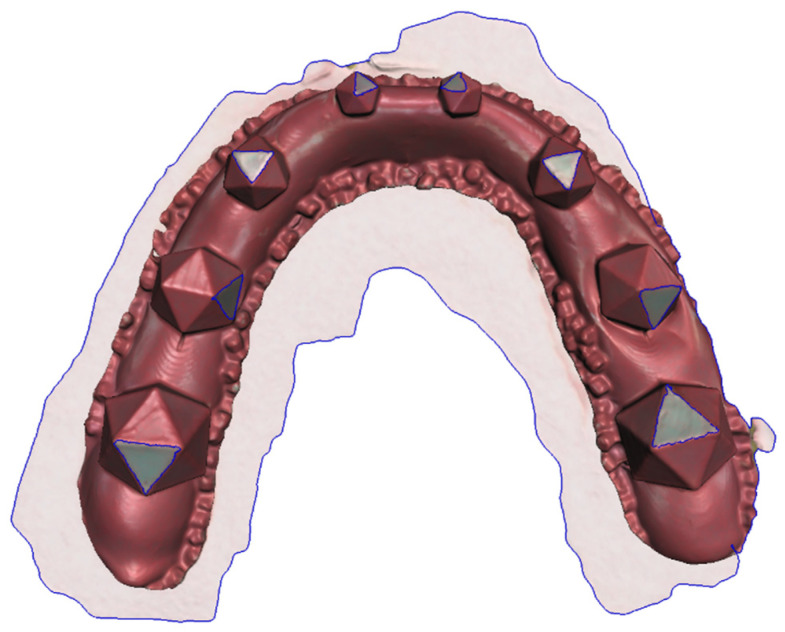
Example of a scanned splint after successful segmentation.

The proposed workflow allowed for the successful completion of all necessary sub-steps. Using the colored powder spray enabled easy and fast scanning, without any artifacts caused by the transparent grinding areas on the splint (Figure 3). Moreover, the clear difference in color between the red splint and the green grinding surfaces allowed for easy segmentation of the grinding surfaces (Figure 4). To assess this statement, the repeatability and accuracy of the scanning procedure were tested as follows. 

The ICC score of 0.998 (95% confidence interval, CI 0.997–0.998; *p* < 0.001), for single measures using a two-way mixed effects model assessing absolute agreement, suggests a high repeatability and reliability of our proposed method. No significant differences between repeated scans and segmentations were detected, suggesting an appropriate repeatability of our approach (F = 1.112; *p* = 0.350).

**Figure 5:**
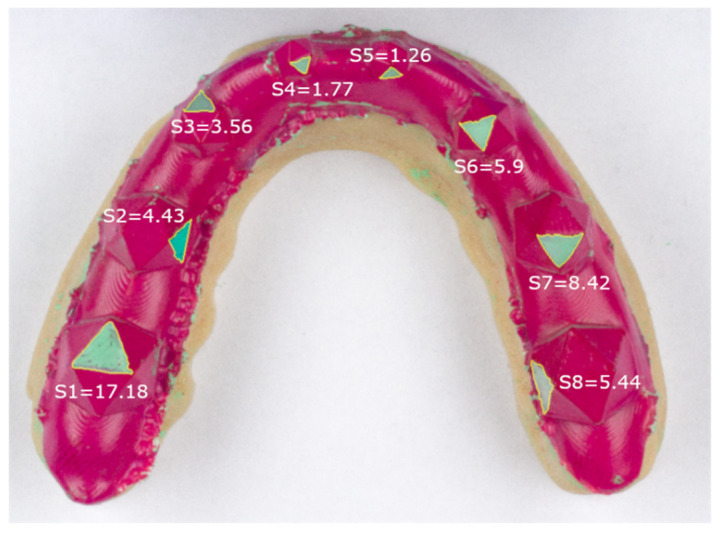
Example of a 2D measurement (Splint 2 shown). Surface area for each grinding surface is reported in mm^2^. While measurements on the same size icosahedrons should be relatively close, stark differences can be seen, e.g., between S1 and S8.

Table 1 reports the mean surface area and standard deviation for each grinding surface for all 18 scans using the 2D and 3D methods. For 2D measurements only a single measurement was completed, while we report the mean over the five repetitions for our new method. Generally speaking, higher standard deviations can be seen for the 2D measurements. Moreover, the independent-samples t-test showed statistically significant differences for all grinding surfaces between surface areas measured in 2D and 3D. Figure 5 shows the results of the 2D measurements for an example splint (Splint 2) and depicts clear differences in grinding size for similarly sized icosahedrons.

The accuracy of the presented method was assessed by reporting the maximum, minimum and mean standard deviations between the five repeated scans of the same splint reported in absolute mm^2^ and relative to the mean size of the grinding area (%; Table 2). The highest maximum standard deviation was 0.63 mm^2^. Generally, a trend for larger absolute variation was found for the measurements of larger grinding surfaces. Taking the size of the grinding surface into account, the largest relative variation was found to be 10.36%. Generally, the relative standard deviation was larger for the smaller grinding surfaces.

## 4. Discussion

The presented study established and reports a novel method for the semi-automatic, quantitative, 3D assessment of grinding surfaces on a colored occlusal splint; a task that, to the best of the authors’ knowledge, has not been accomplished in the previous literature so far. Our measurements suggest a high repeatability and accuracy of the presented method. Overall, the proposed workflow could be a valuable tool for future investigations regarding occlusal variables and has the potential to increase the understanding of various functional, parafunctional and dysfunctional tasks of the masticatory system.

Generally speaking, occlusal splints are a cheap, non-invasive and easy-to-use method to assess the grinding pattern of a patient [14,17]. Consequently, they are a great tool for the initial assessment in bruxism diagnosis [20]. Currently these splints are mostly qualitatively assessed by defining the involved regions of the occlusal grinding patterns (e.g., “canine guided”, “premolar and/ or molar involved”) [16], which limits their diagnostic value. Some quantitative methods have been proposed, but they all use 2D photographs of the splints [14,15]. Those methods so far cannot calculate the grinding area precisely, since the 3D nature of human teeth induces a non-negligible error caused by the 2D projection of a photograph. This error increases with the angle between the 2D projection plane and the grinding facet plane. When models are photographed from above, the largest error can be seen on steep tooth surfaces, e.g., on the canines. By using an optical 3D scanner, we solved this problem and computed accurate 3D shapes.

One major problem during initial testing of the presented method was the detection of the grinding areas during 3D scanning. The patient (or, in our case, the polishing device) grinds off the colored layer on the splint, leaving translucent grinding areas. While these areas are easy to register visually, the translucency of the foil makes them very hard for an optical scanner to detect, which leads to non-repeatable and noisy results, where the scanner sometimes detects the splint and sometimes scans the dental model below the splint. This problem often induces sharp edges and switching of the surface between the level of the cast and the splint, which leads to an overestimation of the grinding surfaces and a generally cumbersome scanning process. We solved this problem by using a colored powder spray with a different color with respect to the splint color. We chose a green spray because it gave good contrast to the red color of the splint and since red and green are well separated in RGB (red, green, blue) color space, we expected this color decision to further improve the segmentation process. This simple and cost-effective solution enabled us to drastically increase the scan quality, while simultaneously reducing scanning time.

To assess the repeatability of our results we scanned each splint five times, segmented the grinding surfaces and compared the differences between the repeated scans. The high ICC of 0.998 detected with the presented method suggests an excellent repeatability. Moreover, this finding was confirmed by detecting no significant differences between the repeated measurements, giving us confidence in the results computed with the proposed method.

In general, the same grinding surface on different splints should be relatively comparable in surface area (e.g., Splint1 S1 and Splint2 S1) with only minor differences caused by the manual grinding process. Moreover, since the triangles on the left and right sides have the same size in our model, differences between the respective surfaces (e.g., S1 and S8) should be minimal. This was indeed true and we could only detect statistically significant differences between the grinding areas of surfaces on differently sized icosahedrons.

To further evaluate accuracy of the measurement method, we investigated the standard deviation of the grinding surfaces between the repeated measurements and compared them to the standard deviation between the grinding surfaces on different splints. Standard deviations were larger between models, compared to repeated measures of the same splint. The largest standard deviation for the repeated measures was 0.63 mm^2^ for surface 8. Relative to the mean grinding area of the surface, the computed standard deviation for surface 8 is equal to 3.73%. As expected, the largest relative difference was found for the smallest grinding surfaces, with 10.36% for Surface 4, which represents an absolute surface of 0.29 mm^2^. These maximum values represent the worst case and when looking at the mean standard deviations for each surface we see values of approximately half the value of these maximums. We think that these relatively small differences suggest an appropriate accuracy for clinically relevant differences in grinding areas.

Additionally, we showcased our novel measurement method by comparing it to the currently used method of assessing surface areas on 2D photographs. Larger standard deviations for the surfaces were found when using the 2D method. As described above, this is due to the fact that, in addition to the standard deviation caused by the actual differences from manual preparation of the grinding areas, an additional variability is included by using grinding facets with different angles with respect to the imaging plane. This can clearly be seen in Figure 5 when comparing S1 and S8. These surfaces are roughly the same size, apart from small variances caused by the manual grinding, but due to the projection error S8 is substantially smaller than S1 when using photographs. By using the presented method this error is drastically reduced. On the other hand, the projection error for S1 is relatively small since the surface is well aligned with the projection plane of the photograph. Consequently, our data show that if a grinding surface with a large angle to the imaging plane is chosen, the surface area was underestimated drastically. As expected, significant differences in grinding area were detected between the two measurement methods (photographs vs. 3D scanning) for all grinding surfaces.

While our study computed convincing results, some limitations remain. Firstly, the occlusal splint used in our study can only assess the direction of the grinding movement, the area and number of occlusal grinding surfaces, but it cannot define the magnitude, frequency and duration of the applied grinding force, which are relevant parameters related to the pathogenesis of TMD [23]. Other splint designs have been proposed that use multiple layers of colored material, inferring some information on grinding force magnitude [14], but some authors have reservations regarding the thickness of these multilayer splints [24]. It was suggested that the thicker splints act in the same way as an actual therapeutic splint and reduce muscle activity, which would make them infeasible as a diagnostic tool. Nevertheless, colored splints have proven to be a valuable first diagnostic tool in bruxism diagnosis [16,17,19,20] and we are confident that our method is transferable to other splint designs. Secondly, we did not compare our optical scans to a different physical measurement of the grinding surface. Optical scanning has been shown to be a valuable and accurate tool in digital dentistry [25,26,27] and is used for various dental applications [28,29]. More specifically, the trueness and precision of the 3D scanner used in this study has been assessed for complete arch scans by multiple previous studies [30,31,32]. Schmidt et al. found a mean deviation of 33.8 ± 31.5 µm. Moreover, Dutton et al. assessed the performance of the Primescan over multiple materials and found a trueness of 17 µm and a precision of 25 µm. Lastly, Ender et al. report a trueness of 33.9 ± 7.8 µm and a precision of 31.3 ± 10.3 µm. Consequently, we do not think that the initial validation of the correctness of the overall geometry has to be proven for our specific study.

Future studies could, for example, focus on the assessment of a potential correlation between occlusal grinding areas in 3D and muscle activity EMG, in order to include additional information on the frequency and magnitude of the grinding events. This could provide important clues to predict diseases of traumatic occlusion and TMJ disorders.

## 5. Conclusions

In conclusion, this study proposes an innovative, fast and cost effective method to support the initial diagnosis of sleep bruxism. Moreover, due to the 3D nature of the presented method, it facilitates the fast and easy quantitative assessment of the surface area of the respective grinding facets. The study results suggest a high accuracy as well repeatability of the proposed method, which will allow for better quantitative assessment and comparison of the grinding areas in future clinical studies. This will potentially help in gathering knowledge and developing better screening and treatment methods for patients in the early stages of sleep bruxism.

## Figures and Tables

**Table 1:** Mean surface area and standard deviation for all 8 grinding surfaces over the 18 prepared splints measured from 2D photographs and 3D scans. *p*-values are reported for independent-samples *t*-test for differences between the measurement methods.

	Surface Area in 2D [mm^2^]	Surface Area in 3D [mm^2^]	*p*-Value
Surface 1	13.2 ± 2.85	17.41 ± 1.2	<0.001
Surface 2	7.61 ± 2.27	11.20 ± 0.61	<0.001
Surface 3	4.90 ± 1.47	6.46 ± 0.61	<0.001
Surface 4	1.98 ± 0.37	2.78 ± 0.29	<0.001
Surface 5	2.01 ± 0.57	2.97 ± 0.32	<0.001
Surface 6	4.80 ± 1.66	6.45 ± 0.71	<0.001
Surface 7	8.80 ± 1.12	10.65 ± 0.44	<0.001
Surface 8	13.56 ± 3.16	16.93 ± 0.86	<0.001

**Table 2:** Standard deviation of repeated measures for each surface over 18 prepared splints.

	Maximum [mm^2^]	Minimum [mm^2^]	Mean [mm^2^]	Percentage of Mean Surface
Surface 1	0.41	0.11	0.25	2.35
Surface 2	0.59	0.14	0.28	5.28
Surface 3	0.48	0.09	0.19	7.43
Surface 4	0.29	0.03	0.12	10.36
Surface 5	0.26	0.08	0.15	8.91
Surface 6	0.35	0.04	0.17	5.38
Surface 7	0.59	0.15	0.28	5.5
Surface 8	0.63	0.15	0.32	3.73

## Data Availability

The data presented in this study are available on request from the corresponding author.

## Appendix A.—Software Workflow

This appendix will briefly describe the software settings and procedures used during the various digital processing and production steps.

Model design: The model is based on the idealized morphology of a gingival arch. We idealized the arch using manual smoothing in the Autodesk^®^ Meshmixer toolkit. Afterwards we selected icosahedrons under Meshmix → Primitives and pulled them onto the appropriate positions on the arch. The offset was kept at 0 and the dimension was used to change the size as described in the Methods section. The icosahedrons were added using the Boolean Union composition mode.

Model 3D Printing: The model was printed in a layer density of 0.05 mm with a total of 413 layers. It was printed within approximately 3 h, consuming 18.48 mL of resin. The object was printed on full rafts, including a raft label and internal supports. Rafts had a touchpoint size of 0.60 mm and a density value of 1. Automated advanced settings included a flat spacing of 5 mm, a slope multiplier value of 1, 5 mm height above raft, 2 mm raft thickness, a Z-compression correction of 0.75 mm and an early layer merge of 0.30 mm. After printing, the object was washed in isopropanol (IPA) for 15 min. Washing was repeated in fresh IPA for another 5 min, followed by drying overnight. The next day, the printed model was cured in a Formlabs^®^ Form Cure for 20 min at 80 °C, following the recommendations of the manufacturer. After curing, all support structures were removed manually.

Splint 3D Scanning: The colored splints were scanned using the dedicated scanning software using the standard parameters. Afterwards the saved .ply files were collected and exported for post-processing.

Splint 3D assessment: .ply files of the scanned splints were opened using the Autodesk^®^ Meshmixer toolkit and the function under Select → Filters → Vertex Color Similarity was used. Back face selection was not enabled and no crease angle threshold was used. Each grinding facet was segmented and separated into its own component (Edit → Separate). Afterwards, each grinding facet was selected and the surface area was computed using Analysis →Stability. Values were computed and collected for all grinding areas of all splints and used for statistical analysis.
